# The use of benzodiazepines and the mental health of women in prison: a cross-sectional study

**DOI:** 10.1038/s41598-023-30604-0

**Published:** 2023-03-18

**Authors:** Fernanda Miranda Seixas Einloft, Luciane Kopittke, Míriam Thais Guterres Dias, Águida Luana Veriato Schultz, Renata Maria Dotta, Helena Maria Tannhauser Barros

**Affiliations:** 1 Programa de Pós-Gradução em Ciências da Saúde, Universidade Federal de Ciências da Saúde de Porto Alegre - UFCSPA, Porto Alegre, Rio Grande do Sul Brasil; 2 Grupo Hospitalar Conceição - GHC, Porto Alegre, Rio Grande do Sul Brasil; 3 Programa de Pós-Graduação em Política Social e Serviço Social, Universidade Federal do Rio Grande do Sul - UFRGS, Porto Alegre, Rio Grande do Sul Brasil; 4 Programa de Pós-Graduação em Psicologia Social e Institucional, Universidade Federal do Rio Grande do Sul - UFRGS, Porto Alegre, Rio Grande do Sul Brasil; 5 Secretaria Estadual da Saúde, Porto Alegre, Rio Grande do Sul Brasil; 6 Departamento de Farmacociências, Universidade Federal de Ciências da Saúde de Porto Alegre - UFCSPA, Porto Alegre, Rio Grande do Sul Brasil

**Keywords:** Public health, Psychology

## Abstract

In this article we assessed the prevalence of benzodiazepine (BZD) use in women before and during imprisonment, as well as its related factors and association with symptoms of anxiety, depression, and posttraumatic stress disorder in a quantitative, cross-sectional, analytical study of regional scope. Two female prisons in the Brazilian Prison System were included. Seventy-four women participated by completing questionnaires about their sociodemographic data, BZD use and use of other substances. These questionnaires included the Generalized Anxiety Disorder-7 (GAD-7), Patient Health Questionnaire-9 (PHQ-9), and Posttraumatic Stress Disorder Checklist–Civilian Version (PCL-C). Of the 46 women who reported no BZDs use before arrest, 29 (63%) began using BZDs during imprisonment (*p* < 0.001). Positive scores for PTSD, anxiety, and depression, as well as associations between BZD use during imprisonment and anxiety (*p* = 0.028), depression (*p* = 0.001) and comorbid anxiety and depression (*p* = 0.003) were found when a bivariate Poisson regression was performed. When a multivariate Poisson regression was performed for tobacco use, the PHQ-9 and GAD-7 scales, BZD use was associated with depression (*p* = *p* = 0.008), with tobacco use (*p* = 0.012), but not with anxiety (*p* = 0.325). Imprisonment increases the psychological suffering of women, consequently increasing BZD use. Nonpharmacological measures need to be considered in the health care of incarcerated women.

## Introduction

Benzodiazepines (BZDs) are usually indicated during the treatment of anxiety disorders, insomnia, muscle spasms, and seizures^1–3^. BZDs are among the most prescribed drugs, with the number of prescriptions increasing in the last two decades^4^. In the USA, prescriptions for BZDs have increased threefold or more in the last decade, particularly for long-term users^4–6^. The rates of BZD use are 5.2% of adults in the US^2^, 4% in Canada^7^, and 7.5% in France^8^. In the Brazilian general population, 3.6% of individuals use BZDs, and 7.8% of individuals have mental health problems^9^. However, little information on prevalence rates and patterns of BZD use in female prisoners is available in the literature^10^.

Hassan et al.^11^ discuss the surprisingly high prevalence of prescribed psychotropic drugs, including BZDs, for female prisoners compared to that of male prisoners and women in the general population. These authors found a higher prevalence of hypnotic and anxiolytic prescriptions for female prisoners (with diazepam prescriptions comprising 50% of prescriptions). The prevalence of prescriptions was 7.9% in female prisoners but 1.0% in male prisoners and 2.5% in the general female population. For female prisoners, 85.7% of these prescriptions represent a valid indication of these drugs and 98.3% represent a valid dose^11^.

A worrying emerging reality is the significant increase in female incarceration rates internationally^12^. In Brazil, the general incarceration rate increased by more than 150% between 2000 and 2017, which calls for increased attention of researchers and suggests the need for more research in prisons^13,14^.

An important problem identified in female prisoners is the prevalence of psychiatric disorders^15,16^. Incarcerated women show higher prevalences of most illnesses, especially depressive disorders, posttraumatic stress disorder, and substance use disorders^17^.

In addition to psychiatric disorders, there has been an increase in drug use disorders in prisons during the last three decades^18^. Two-thirds of sentenced prisoners (63%) meet criteria for addiction or abuse of marijuana/hashish, cocaine/crack, heroin/opiates, depressants, stimulants, methamphetamines, hallucinogens and inhalants^19^. Higher rates of lifetime substance use disorder are found in female prisoners than in females in the general population (25.2% vs. 1.6%)^20^. An Irish study found a 17.2% rate of alcohol use disorder and 62.6% rate of substance use disorder^21^ in a female prison population.

In this study we aim to verify the prevalence of BZD use and other substance use in incarcerated women before and during imprisonment as well as the association of BZD use with symptoms of anxiety, depression, and posttraumatic stress disorder (PTSD).

## Methods

### Study design

This study is quantitative, cross-sectional, and analytical, with regional coverage. It is a secondary analysis of data from the PPSUS Project, “Incarcerated women: Context of violence and needs arising from drug use”, EFP_00013968^22^. After an initial analysis of the data, our attention was drawn to the significant increase in BZD use by women during incarceration, which motivated the present study.

### Study population and inclusion criteria

The population consisted of women from the Prison System of Porto Alegre and the Metropolitan Region of Porto Alegre. Two prisons were selected that had a basic health unit and comprised a total of 502 women, in 2019^23^. Seventy-four women over 18 years old who had served a sentence for at least 6 months in closed conditions were randomly selected. The women who agreed to participate in the research signed an informed consent form.

### Data collection methods

The researchers participating in the data collection were trained so that they could administer the questionnaires in the same way. For the data collection, self-assisted tablet interviews were conducted through an offline system, Research Electronic Data Capture (REDCap)^23^, using a structured questionnaire with closed questions about sociodemographic characteristics, information about time and cause of incarceration, use of BZD and other drugs (tobacco, alcohol, marijuana, snorted cocaine, crack) and mental health. These questions were taken from the following validated scales: the Posttraumatic Stress Disorder Checklist–Civilian Version (PCL-C)^24^, the Patient Health Questionnaire-9 (PHQ-9)^25^, and the Generalized Anxiety Disorder scale-7 (GAD-7)^26^.

BZDs were the substance of interest in this study, as they are a subclass of one of the ten classes of drugs related to relevant disorders, according to the DSM-5^27^ Self-reported data on BZD use including the frequency of use and whether the use was under prescription were collected for two time points: describing use before being arrested and in the last 6 months after their arrest. For the frequency of use, responses were grouped into the following categories and subcategories: more than once per week (every day and more than once a week), from once a week to occasional use in 6 months (once a week to more than once every 6 months), and approximately once in 12 months (once every 6 months, more than once a year, once a year, once in a lifetime). Nondichotomous variables were grouped into categories of race (White or Caucasian and other races, including Asian, Hispanic or South Asian, Black (quilombola), and Black (nonquilombola); level of schooling (less than 8 years of schooling and secondary/higher education) and marital status (single, which included single and separated/divorced/widowed women, and married, which included women who were married, in a stable relationship or with a steady partner). Two time points, before and during imprisonment (the 6 month period following arrest), were considered during data collection on the type of drug used (tobacco, alcohol, marijuana, cocaine snorted, crack) and frequency of use. The mental health assessment of female prisoners considered the period of 6 months after imprisonment. The questionnaires used were the (PCL-C with a cutoff of 50^24^; the PHQ-9 with a cutoff of 10^25^; and the GAD-7 with a cutoff of 10^26^. The scores were considered “Negative” or “Positive” based on the cutoff values.

### Statistical analysis

Statistical analyses were completed using SPSS, version 20.0. A significance level of *p* ≤ 0.05 was adopted. The prevalence for the study’s categorical variables was determined. To describe the age variable, the mean and standard deviation were used. A multicollinearity test was performed between the PHQ-9 and GAD-7 to certify that there would be no overlapping variables compromising the results. To assess the change in BZD use and other substances before and during imprisonment, the McNemar chi-square test was used. To assess the change in the frequency of use, the McNemar-Bowker chi-square test was used. A simple robust Poisson regression was performed to assess the association of different risk factors with BZD use (95% confidence interval). After this analysis, variables with *p* < 0.20 (PHQ-9, GAD-7, tobacco use with BZD use) were included in the multivariate Poisson regression^28^.

To compare the overall use of BZDs in female prisoners before and during imprisonment, discordant pairs were used (39.2% of those who did not use BZDs before and started using BZDs during imprisonment and 5.4% who used BZDs before and stopped using BZDs during imprisonment), with a power of 99%.

### Ethics declarations

All methods were performed in accordance with the relevant guidelines and regulations. The present study was approved by the Ethics in Health Research Committee of the Psychology Institute of the Universidade Federal do Rio Grande do Sul (UFRGS) (#2.832.322).

## Results

The following characteristics of the studied population were found: predominance of young inmates, with a mean age of 37.8 to 38.9 years among nonusers and users of BZDs; 43 (58.1%) White or Caucasian women and 31 (41.9%) women of other races (Asian, Black (quilombola), and Black (nonquilombola). Regarding education, 53 (71.6%) women achieved less than 8 years of study, and 21 of them (28.4%) had completed high school or pursued a university degree (complete or incomplete). Fifty (67.6%) women were married (including those with a stable union or steady partner), while 24 (32.4%) reported being single, separated, divorced or widowed. Sixty-six (89.2%) reported being mothers, and 8 (10.8%) did not have children. Forty-five women (60.8%) reported that they were responsible for family income, and 28 (37.8%) reported that they were not.

At the time of data collection, the prison time interval of the women participating in this research was 7 months to 138 months, with an average prison time of 32.65 months. According to the criminal classification, 32 women (43.2%) were arrested for crimes related to drugs, 21 (28.4%) for crimes against people, 1 (1.4%) for crimes related to weapons, 10 (13, 5%) for crimes against sexual dignity and 12 (16.2%) women were arrested for crimes against property.

There was a relationship between tobacco use and BZD use. An association between BZD use and sociodemographic variables was not found in prison (Table 1).

**Table 1:** Relationship of sociodemographic variables and use of tobacco with the in-prison use of benzodiazepines (last 6 months) (*n* = 74).

Sociodemographic variable	Use of benzodiazepines *n* = 53	Nonuse of benzodiazepines *n* = 21	PR	CI (95%)	*p*-value
Age	38.9 (± 10.8)	37.8 (± 11.7)	1.003	0.989–1.016	0.704
Self-reported race/color					
White or caucasian	34 (79.1)	9 (20.9)	1.318	0.947–1.833	0.101
Other race^a^	18 (60)	12 (40)	1.0		
Level of schooling					
Up to 8 years for schooling^b^	37 (69.8)	16 (30.2)	0.916	0.680–1.234	0.565
High school/College^c^	16 (76.2)	5 (23.8)	1.0		
Marital status					
Single^d^	37 (74)	13 (26)	1.0	0.650–1.250	0.532
Married^e^	16 (66.7)	8 (33.3)	0.901		
Maternity					
No	5 (62.5)	3 (37.5)	0.859	0.492–1.5	0.594
Yes	48 (72.7)	18 (27.3)	1.0		
Responsibility over family income					
No	20 (71.4)	8 (28.60)	1.0	0.727–1.306	0.860
Yes	33 (73.3)	12 (26.7)	0.974		
Tobacco use					
No	13 (50)	*13 (50)*	1.0	1.112–2.498	0.013*
Yes	40 (83.3)	8 (16.7%)	1.667		

*A bivariate Poisson Regression was performed; ^a^ [Asian, Black (quilombola), and Black (nonquilombola)]; ^b^ (Illiterate; 1st to 4th grade of elementary school- incomplete or complete, 5th to 8th grade of elementary school—incomplete or complete); ^c^(Incomplete or complete); ^d^Single (including those separated or divorced or widowed); ^e^Married (including those with a stable union or steady partner), the age variable was described with mean and standard deviation (± SD), CI confidence interval, PR prevalence ratio. Significant values are in italics.

**Figure 1:**
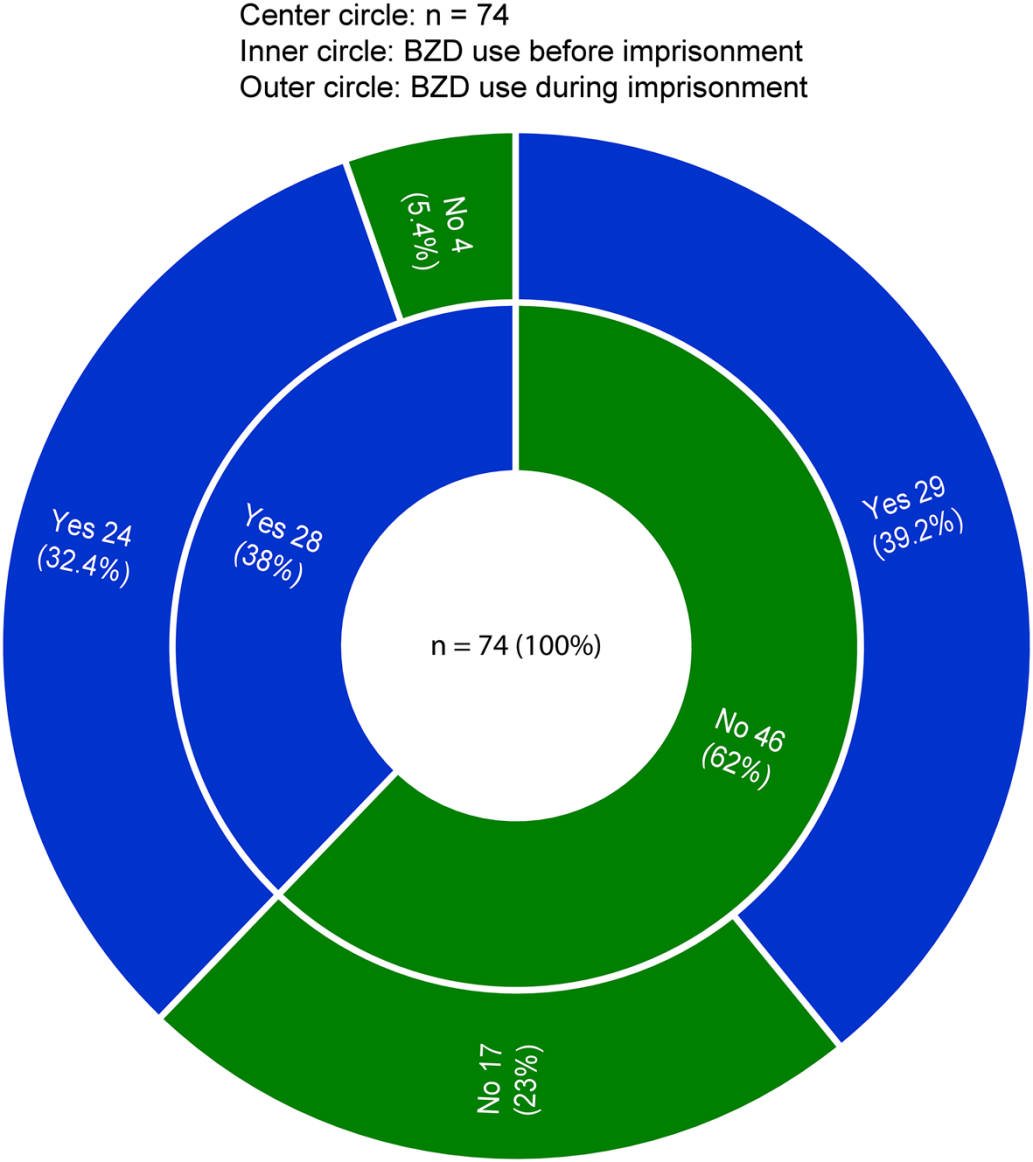
BZD use by women before and during imprisonment.

Of the 74 women inmates, 28 (38%) women used BZDs, and 46 (62%) women did not use BZDs before imprisonment. Of the women who used BZDs before imprisonment, 24 (32.4%) continued using BZDs, and 4 (5.4%) stopped using BZDs during imprisonment. Of the 46 (62%) women who did not use BZDs before prison, 17 (23%) continued to not use BZDs, while 29 (39.2%) started using BZDs during imprisonment. Of the 46 women who did not use BZDs, a significant proportion 29 (63%) started to use BZDs (*p* < 0.001) (Fig. 1).

**Figure 2:**
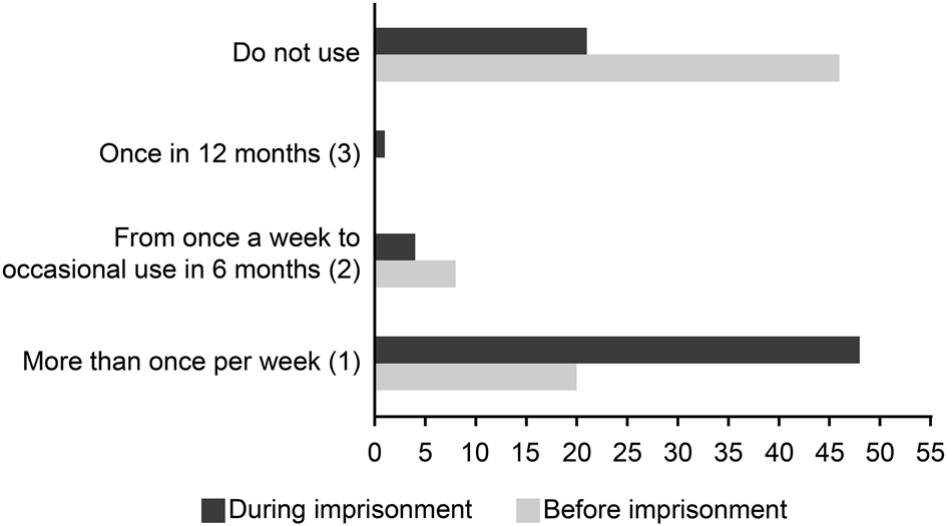
Frequency of BZD use by women before and during prison.

The participants showed a substantial increase in their frequency of BZD use. Before imprisonment, 20 women (27%) reported frequent use of BZDs (more than once a week), while 46 (62.2%) women reported not using BZDs. During imprisonment, 21 (28.4%) of the 46 women who had not used BZDs continued to not use BZDs, but a significant proportion of them, 25 (54%), started using BZDs very frequently (more than once a week), with a *p* value < 0.001. The 48 (64.9%) women who used BZDs more than once a week during imprisonment comprised the women who used BZDs more than once a week before imprisonment (n = 20; 27%) + women who did not use BZDs before imprisonment and started to use BZDs during imprisonment (n = 25; 33.7%) + the number of women who used BZDs before imprisonment at a frequency of once a week to occasional use in 6 months (n = 3; 4.2%) (Fig. 2).

We also quantified prevalence rates for the current use of tobacco (64.9%), marijuana (10.9%), snorted cocaine (8.2%), crack (4.1%) and alcohol (1.4%). A statistically significant decrease in the use of alcohol, marijuana, snorted cocaine and crack was observed when comparing current use during incarceration (including women who previously used these substances and continued to use them in prison and those who did not previously use them and started to use them during incarceration) with previous use (Table 2).

**Table 2:** Use of other substances by female inmates, in two Penitentiaries in the Metropolitan Region of Porto Alegre, before and during imprisonment (*n* = 74).

	Used before and continued now n (%)	Did not use before and started to use n (%)	Used before and quit n (%)	Never used (%)	*p* value
Tobacco	42 (56.8%)	6 (8.1%)	5 (6.8%)	21(28.4%)	> 0.999
Alcohol	1 (1.4%)	0 (0.0%)	41 (55.4%)	32 (43.2%)	< 0.001
Marijuana	7 (9.5%)	1 (1.4%)	14 (18.9%)	52 (70.3%)	0.001
Snorted cocaine	5 (6.8%)	1 (1.4%)	18 (24.3%)	50 (67.6%)	< 0.001
Crack	3 (4.1%)	0 (0.0%)	18 (24.7%)	52 (71.2%)	< 0.001

*p value of < 0.001 obtained by McNemar chi-square Test.

Considering the similar effects on the central nervous system (CNS) of BZDs and alcohol (a CNS depressant agent)^27^, we analyzed the use of alcohol before incarceration and the current use of BZDs. The results did not show a statistically significant different in alcoholic beverage use before and BZD use after (p = 0.637).

The rates of PTSD, depression, and anxiety based on the mental health questionnaires used in this study are shown in Table 3. More than 80% of the women who had “positive” scores for PTSD, anxiety and depression reported use of BZDs. In the Poisson regression analysis of the mental health assessment scales (GAD-7, PHQ-9, and PTSD) and BZD use, it was found that of the 22 women with PTSD (positive PCL-C classification), 18 women (81.8%) were using BZDs; however, there was no statistically significant relationship between PCL-C classification and BZD use (*p* = 0.161). Regarding the GAD-7, of the 45 women who had anxiety based on a positive GAD-7 classification, 37 (82.2%) reported using BZDs during incarceration with *p* = 0.028, indicating a significant relationship between GAD-7 classification and BZD use. When analyzing the PHQ-9 scale, a total of 33 participants had depression based on a positive PHQ-9 classification, of which 30 (90.9%) used BZDs with *p* = 0.001. A significant relationship was found (p = 0.003) for women who had comorbid anxiety and depression and reported using BZDs (Table 3).

**Table 3:** Association of BZD use during imprisonment with GAD-7, PHQ-9 and PCL-C scales (*n* = 74).

Scales	Score^a^	Use of BZD *n* (%)	Nonuse of BZD *n* (%)	PR	CI (95%)	*p* value
Anxiety (GAD-7)	Negative	16 (55.2)	13 (44.8)	1.0	1.045–2.126	0.028*
	Positive	37 (82.2)	8 (17.8)	1.490		
Depression (PHQ-9)	Negative	23 (56.1)	18 (43.9)	1.0	1.211–2.169	0.001*
	Positive	30 (90.9)	3 (9.1)	1.621		
Anxiety and depression (GAD-7 and PHQ-9)	Negative	27 (60)	18 (40)	1.0	1.142–1.955	0.003*
	Positive	26 (89,7)	3 (10.3)	1.494		
Post-traumatic stress disorder (PCL-C)	Negative	35 (67.3)	17 (32.7)	1.0	0.925–1.598	0.161
	Positive	18 (81.8)	4 (18.2)	1.216		

A bivariate Poisson Regression was performed; ^a^Score values according to each scale are: PCL-C Negative (< 50), Positive (> = 50); GAD-7 Negative (< 10), Positive (> = 10); PHQ-9 Negative (< 10), Positive (> = 10), and should be considered Negative when there is no indication of the disorder and Positive when there is an indication of the disorder in question. CI confidence interval, PR prevalence ratio. Significant values are in italics.

Before incarceration, 100% of BZD use was prescribed. During imprisonment, the rate of BZDs used that was prescribed dropped to 88.7%; 6 women (11.3%) self-reported the use of BZDs without a prescription.

When a multivariate Poisson regression was performed with BZD use, PHQ-9 and GAD-7 scales, and tobacco use, BZD use was associated with the PHQ-9 scale (*p* = 0.008), and tobacco use (*p* = 0.012), but not the GAD-7 (*p* = 0.325) (Table 4).

**Table 4:** Relationship of BZD use during imprisonment with the PHQ-9, GAD-7 scales, and tobacco use (*n* = 74).

Scale	Score	*n* = 74* (%)	PR	CI (95%)	*p* value
Anxiety (GAD-7)	Negative	29 (39.2)	1.0	0.848–1.643	0.325
	Positive	45 (60.8)	1.181		
Depression (PHQ-9)	Negative	41 (55.4)	1.0	1.103–1.898	0.008*
	Positive	33 (44.6)	1.447		
Use of tobacco	No	26 (35.1)	1.0	1.106–2.271	0.012*
	Yes	48 (64.9)	1.584		

A multivariate Poisson regression was performed; * Dependent variable—BZD use; Score values according to each scale are: PCL-C Negative (< 50), Positive (> = 50); GAD-7 Negative (< 10), Positive (> = 10); PHQ-9 Negative (< 10), Positive (> = 10), and should be considered Negative when there is no indication of the disorder and Positive when there is an indication of the disorder in question. CI confidence interval, PR prevalence ratio.

## Discussion

The main results are as follows: (a) the prevalence of BZD use by women more than doubles in the first months of incarceration; (b) BZDs are used more frequently during imprisonment than before imprisonment; (c) Positive scores for PTSD, anxiety, and depression had high prevalence rates; (d) There is an association between BZD use by women in prison with anxiety, depression and comorbid anxiety and depression; (e) Women who use BZDs have a statistically significant likelihood of having a positive score for depression but not for anxiety; (f) tobacco use increased during incarceration and the use of other substances decreased; (g) no association was found between BZD use in prison and alcohol use before prison.

Most data on sociodemographic characteristics are in accordance with the population of incarcerated women in Brazil^29^; these women are quite young, have a low educational attainment, are not married and are mothers of one or more children. The main difference is that there is a predominance of White women included in the current study, while in the Brazilian female prison population, 48.04% are mixed-race and 15.51% are black. The three southern Brazilian states have a greater number of incarcerated White women, with 63% of the incarcerated population being White or Caucasian^30^, which follows the general profile of the population of Rio Grande do Sul that is related to immigration waves from the nineteenth century that predominantly included European immigrants^31^. Considering that almost 90% of these women were mothers, it is noteworthy that the interruption of close relationships, especially separation from children and abandonment by friends, are factors that worsen mental suffering in female prisoners^32,33^. The breakdown of the family unit after imprisonment has worse consequences for women than men^32^.

This study found an increase in the prevalence and frequency of BZD use in women during imprisonment. It is recognized that nonincarcerated women have high rates of BZD use^2,4,27,34,35^, and the increase in BZD use, both in prevalence and in frequency of use by women during imprisonment, might be related to stressors and anxiogenic factors related to incarceration itself^33,36^. Most prisons are adapted spaces^36^ that are uncomfortable, overcrowded, noisy, and busy, with poor infrastructure and sanitary conditions^10,33,37,38^. Women report feelings of degradation, humiliation and objectification^36^, with consequent changes in their self-conception^32^. Sadness, loneliness, abandonment, anger, idleness, anxiety, stress, depression, and changes in sleep patterns are factors that affect the mental health of women in this context^33^.

In addition to their calming function, BZDs provide a feeling of disconnection or escape from reality and a change in temporal perception (with BZDs, time seems to pass faster), motivating incarcerated women to use them^33,36,39^. The reported increase in use and frequency of use in the first months during imprisonment needs to be thoroughly considered in addition to guidelines for BZD prescription and the risk of misuse^40^.

A high prevalence of mental health conditions was found here, as in other studies in the international literature^12,32,33,41–43^. The mental health of women in prison is more affected than their physical health due to factors that are socially inherent to the gender, namely, responsibility for their families in combination with isolation and lack of social support^32^. Here, we show that approximately one-third of female inmates presented with PTSD, similar to other studies^44^, reflecting the extent to which incarceration per se and its consequences are traumatic. Previous studies have shown that female inmates have higher prevalence rates of mental health conditions than male inmates^44^, and there are significant associations between PTSD and other disorders (depression, anxiety, and substance use)^45^. The prevalence of depression among inmates of both sexes is approximately 40%^46^, but it is almost 50% among older female inmates^47^. The rate of depression in older female inmates is similar to the rate observed here, although this group is composed of younger women. One-third of the inmates interviewed reported anxiety and depression. In prisons, anxiety prevalence rates range between 19.1^38^ and 43% in older female inmates^47^, which are much lower than the rates observed here, with 60.8% of the inmates having a positive GAD-7 classification. The potentially traumatizing environment of prison^12^ does not provide the emotional or psychological support these women need. BZD use could be avoided if evidence-based psychological care were properly offered.

In Spain, in addition to symptoms related to anxiety and depression, consumption of alcohol and other drugs is frequent in female inmates^48^. An American report found higher rates of substance use in female prisoners (47%) than in male prisoners (38%)^19^. Incarcerated women have a 20% rate of alcohol use disorders and a higher, 51%, prevalence rate of illicit drug use disorders^18^. Much lower prevalences were found in our study, especially for the use of alcohol and other drugs. This result seems to be more related to the control system in Brazilian prisons, which limits access to drugs, than to effective care responses for these women. An estimate of the prevalence of nicotine use during imprisonment in low- and middle-income countries ranged from 5 to 87%, with an overall estimate of 56%^16^. A Swiss study found 78.3% rates of women smoking in prison^49^. The high prevalence of smoking in prison found in our study (65%) and in the international literature suggests that smoking-related policies need careful review. Our study did not find a relationship between alcohol use before imprisonment and BZD use during imprisonment, although pharmacological treatment with BZDs represents the gold standard for alcohol withdrawal syndrome treatment^50^.

One of the limitations of this study is the relatively small number of participants, emphasizing the need to expand the studied population. It is possible that a considerable number of women at high risk of BZD misuse or abuse did not participate in the survey, which may have led to underestimation of the prevalence of BZD use. The results of this research were obtained from a sample of women in two prisons in the southern region of Brazil and should be applied with caution in other locations. Brazilian female prisons may differ in their structure from prisons in other locations. The absence of data on the use of other psychotropic medications is another limitation of this study. Another aspect is that the prevalence of BZD use and frequency of use was based on self-reported responses, which may introduce a memory bias.

## Conclusion

This study emphasizes the urgency of discussing the precise indication and rational BZD use in women in prison. Current guidelines indicate the risks of inadvertently prescribing BZDs. This reinforces the importance of structuring strategies for the rationalization of use, ranging from indications based on scientific evidence to the prioritization of nonpharmacological interventions for the treatment of female inmates.

Prison environments and women’s mental health needs are complex. Female inmates require care that begins with specific knowledge of women and their vulnerabilities. This study confirms the importance of interdisciplinary research that includes different perspectives for the structuring of public health policies.

It is noteworthy that researching BZD use in incarcerated women reinforces the importance of study designs that address phenomena as they happen in real life. It is expected that these results will help in the structuring of public health policies aimed at women in prison. The understanding of the prevalence of mental suffering and the increase in the use and frequency of BZDs and tobacco found in this study may ultimately contribute to the construction of effective surveillance and monitoring strategies for this population, to the organization of the provision of services and to the recognition of the need for the incorporation of interdisciplinary therapeutic resources in the treatment of PTSD, anxiety, and depression in prisons.

## Data Availability

Women prisoners constitute a vulnerable population. Requests for materials and data relating to this study should be addressed to F.M.S.E.
